# Effects of person-centered care at the organisational-level for people with dementia. A systematic review

**DOI:** 10.1371/journal.pone.0212686

**Published:** 2019-02-22

**Authors:** Lynette Chenoweth, Jane Stein-Parbury, Samuel Lapkin, Alex Wang, Zhixin Liu, Anna Williams

**Affiliations:** 1 Centre for Healthy Brain Ageing (CHeBA), Faculty of Medicine, The University of New South Wales Sydney, New South Wales, Australia; 2 Faculty of Health, University of Technology Sydney, New South Wales, Australia; 3 School of Nursing, Faculty of Science, Medicine and Health, University of Wollongong, South Western Sydney Campus, New South Wales, Australia; 4 Stats Central, The University of New South Wales Sydney, New South Wales, Australia; 5 School of Nursing, Faculty of Medicine, The University of Notre Dame Sydney, New South Wales, Australia; Nathan S Kline Institute, UNITED STATES

## Abstract

The aim of the systematic review was to determine the effectiveness of organizational-level person-centered care for people living with dementia in relation to their quality of life, mood, neuropsychiatric symptoms and function. ALOIS, the Cochrane Dementia and Cognitive Improvement Group Specialised Register databases, were searched up to June 2018 using the terms dementia OR cognitive impairment OR Alzheimer AND non-pharmacological AND personhood OR person-centered care. Reviewed studies included randomized controlled trials (RCTs), cluster-randomized trials (CRTs) and quasi-experimental studies that compared outcomes of person-centered care and usual (non-person-centered) care, for people with a diagnosis of dementia. The search yielded 12 eligible studies with a total of 2599 people living with dementia in long-term care homes, 600 receiving hospital care and 293 living in extra-care community housing. Random-effects models were used to pool adjusted risk ratios and standard mean differences from all studies; the findings were assessed followed the PRISMA guidelines and GRADE criteria. Statistical heterogeneity was assessed using the I^2^ method and Chi^2^ P value; studies with low statistical heterogeneity were analyzed using a random-effects model with restricted maximum likelihood estimation in R. Analyses of pre/post data within 12 months identified: a significant effect for quality of life (standardized mean difference (SMD) 0.16 and 95% CI 0.03 to 0.28; studies = 6; I^2^ = 22%); non-significant effects for neuropsychiatric symptoms (SMD 0.06, 95% CI -0.08 to 0.19; studies = 4; I^2^ = 0%) and well-being (SMD 0.15, 95% CI -0.15 to 0.45; studies = 4; I^2^ = 77%); and no effects for agitation (SMD -0.05 (95% CI -0.17 to -0.07; studies 5; I^2^ = 0%) and depression (SMD -0.06 and 95% CI -0.27 to 0.15, studies = 5; I^2^ = 53%). The evidence from this review recommends implementation of person-centered care at the organizational-level to support the quality of life of people with living with dementia.

## Introduction

Fifty million people world-wide presently live with dementia [1] and approximately 65.7 million people are projected to be associated with the disorder by 2030 [2]. Encompassing a range of neurocognitive disorders, dementia is distinguished by progressive decline in cognition and impairment in function. Providing care for people with living with dementia is often exhausting and stressful for families and often at a late stage of the disorder, the person will require formal care support, such as long-term residential care [3]. There is growing awareness among healthcare service providers that person-centred care (PCC) is the preferred model of dementia care [1,3,4]. PCC focuses on supporting the person’s remaining abilities, rather than on the losses occurring [4,5], and it recognises the importance of knowing the individual’s history, personality and preferences, bringing the person into shared decisions on their care [6,7], and customizing care and lifestyle support accordingly [7].

The social-psychological theory of personhood in dementia [7] provides the basis for the PCC approach; it proposes that people exist in a social, relational context, and that positive and enriching interpersonal relationships can prevent the disabling effects of dementia and promote a sense of well-being [7,8,9]. Since the person’s life experiences are constructed by the social-psychological milieu, this can often have a more significant effect on the person than the illness itself, by influencing their relationships with caregivers [10]. Kitwood developed a set of guiding PCC principles [7] to help caregivers support the person’s well-being, which include: creating and strengthening a positive relationship with the person through warm and accepting human contact; communicating respectfully, valuing and honoring the person; treating the person as a sentient and unique human being by valuing their innate nature; assisting the person to retain their remaining strengths; viewing the person’s world from their own perspective; and enabling the person to feel socially confident and maintain emotional attachments [7,8,9].

Multiple sources, including international policy [1, 3], dementia advocacy groups [10] and national Dementia Strategies, for example in the UK [11] and Australia [12] advocate PCC for people living with dementia. This recommendation is based on the evidence that PCC can reduce the incidence of clinical issues, such as agitation [13] and delirium [14], and it can help with deprescribing of psychotherapeutic medicines [15]. Despite evidence of the effectiveness of PCC, a pervasive challenge in applying the PCC across healthcare services is the construction of dementia as a master status through use of labelling and social positioning, in which the person’s dependency and a lack of autonomy are expected by caregivers. When this attitude prevails in healthcare services, care becomes focused on compensating for the loss of functional and cognitive abilities rather than supporting remaining abilities [16]. This attitude extends to normalizing the presence of agitation and other neuropsychiatric symptoms in dementia, attributing dementia as the cause rather than the care context [11]. These prevailing caregiver attitudes serve to distance the caregiver from the person living with dementia, resulting in their diminished personhood [7, 11]. These non-PCC practices largely occur because caregivers are unclear on how to integrate PCC principles within existing healthcare services that are constrained by established ways of delivering health care (micro and macro levels) and because of task-focused workplace cultures [17, 18].

Redressing these limitations demands reconceptualizing the organization’s approach to dementia care, instituting enabling and proactive organizational support, attending to managerial and senior staff leadership, and providing targeted staff education, training, direct supervision and oversight of PCC [19,20,21]. Organizational-level provision of PCC requires a top down, bottom up approach; leadership is required from the top and staff caregivers must be equipped with PCC knowledge, skills and attitudes. While individual caregivers need to be skilled in the delivery of person-centred care, the entire organisation must also be supportive. Organisational leaders need to demonstrate commitment and support by articulating a clear vision of PCC, allocating sufficient resources that enable PCC implementation, and establishing and exhibiting values of organization-wide respect, empowerment and choice for the person, their family/advocates and their caregivers [4,5,6,20,21,22]. Leaders need to demonstrate attitudes and behaviours that support PCC by placing relationships before tasks in the care planning and delivery and enabling caregivers to balance the values and wishes of the person (and their family/advocates) with organizational values, in order to provide personalized care delivery in daily practice [4,5,6,17,18, 20–23]. In addition, a shared governance system needs to be established such that caregivers become part of the decision-making process by seeking their input and feedback on decisions regarding changes to policies and procedures, redesigning the care environment, and determining the effect of changes on daily workflow [5,6,21,22,23]. Finally, the physical environment needs to be adapted in order to support the person’s right to privacy, maximise the person’s independence, enable the person to make the best use of their capabilities, provide the person with opportunities to participate in community life and maintain emotional connections, and empower the person to feel psychologically secure, and physically comfortable and safe [4,5,6,23].

In establishing this level of organizational support of PCC, a coordinated approach is needed to communicate the common values of PCC across the healthcare organization. This requires a shift in the organization’s strategic direction, whereby the work culture, leadership and support to individuals within the care relationship must align with person centered values [7,8,9, 21,23]. The organization’s executive and managers must also create a climate of understanding and acceptance among staff that the change process will take some time and will require a great deal of commitment and effort by all members of the organization [20]. Healthcare providers, managers and direct caregivers will need to be part of the change process by embracing a reconceptualized future for the organization, the care recipients and themselves, and by investing in staff who are future oriented [21,23]. To achieve this, organizational systems must facilitate clear communication on PCC requirements, provide capacity for speedy problem solving among all team members [19, 21, 23], encourage new ideas and incorporate an effective way of discovering what is/not working for the person with dementia and respond accordingly [21,22].

A coordinated and sustained cultural and structural transformation supporting PCC requires the healthcare service to focus on respectful and positive relationships between caregivers, people living with dementia and their families, improved capacity of caregivers to provide PCC through the development of knowledge and skills, supporting the dignity and autonomy of the person living with dementia, and promoting collaboration and team work in delivering care that aims for well-being [18, 22,23]. These requirements are encapsulated in the VIPS framework [24] which provides guidance on organizational-level implementation of PCC, paying attention to the following four key elements:

1. Valuing: valuing service user and service staff.
  The organization’s mission statement identifies provision of a person-centred service; Human resources management ensure staff feel valued by their employer; Management practices are empowering to direct service staff; Management supports training and development for staff to be skilled in person-centred care; Management provides supportive and inclusive physical and social environments for people with cognitive disability; and Continuous quality improvement mechanisms are in place that are driven by knowing and acting upon the needs and concerns of service users.
2. Individualized Care: Treating people as individuals.
  Strengths and vulnerabilities of service users are recognised across a wide range of needs; Care plans are individualized that reflect a wide range of strengths and needs; Individual care plans are reviewed on a regular basis; Service users have their own personal clothing possessions for everyday use; Individual likes and dislikes, preferences and daily routines are known about by direct care staff and are acted upon; Care staff are aware of basic individual life histories and key stories of proud times, and are used regularly; and A variety of activities are available to meet needs and abilities of all service users.
3. Personal perspective: looking at world from perspective of person with dementia.
  Service users are asked for their preferences, consent and opinion on a day-to-day basis; Staff show the ability to put themselves in the position of the person they are caring for and think about decisions from their point of view; The physical environment (e.g. noise, temperature) is managed on a day-to-day basis to help people with dementia feel at ease; Physical health needs of people with dementia, including pain assessment, sight and hearing problems, are given due attention; ‘Challenging behaviour’ is analysed to discover the underlying reasons; and Rights of individuals are protected in situations where actions of an individual are at odds with the safety and well-being of others.
4. Social environment: the total human relationship environment, including staff /service user relationships.
  Staff help all service users to be included in conversations and help them to relate to others, despite cognitive and mental ability; Service users are treated with respect, with an absence of people being demeaned by ‘telling-off’ or labelling; There is an atmosphere of warmth, acceptance and comfort to service users; Service users’ fears are taken seriously; Service users are not left alone for long periods in emotional distress; Staff help service users, including those with cognitive disabilities, to be active in their own care and other activities of daily living, and not treat them as objects with no feelings; and Service users are encouraged to use local community facilities and to encourage people from local community to visit regularly.

Two validated assessment tools can be used to assesses aspects of the organizational requirements to support PCC; the Short Observation Framework for Inspection, version 2 (SOFI 2) [25] and the 76-item Person-Centred Environment and Care Assessment Tool (PCECAT) [26]. The SOFI 2 [25] is one component of the Care Quality Commission (CQC) inspection toolkit used to assess the quality of social care services according to the VIPS framework [24], chiefly in the United Kingdom. CQC inspectors use SOFI 2 [25] to observe the mood and engagement of people using services and the quality of staff interactions, and other aspects of care practice during their observations. This assessment tool, therefore, focuses more attention at the individual level of care services. By contrast, the PCECAT [26] assesses both the organisational elements that support PCC for people living in aged care homes, as well as service quality according to PCC principles. It aligns with care home accreditation standards in the Australasian region. The PCECAT [26] includes items on organisational characteristics such as staffing numbers, mix, skills and education in PCC, the organisational culture, care delivery systems, service quality systems, social and therapeutic activity programs, interpersonal relationships and interactions between individuals and caregivers, and the physical layout and design. The PCECAT [26] enables care service providers and assessors to evaluate whether and how PCC is occurring at the individual level and how PCC is being supported at the organizational-level.

## Methods

### Review objective

With reference to the requirements of the four VIPS [24] elements, the objective of the review was to determine the effectiveness of organization-level PCC interventions for people living with dementia, in relation to: reduction of neuropsychiatric symptoms, including agitation; changes in mood, including depression and well-being; improvement in quality of life; improvement in activities of living; alterations in the use of restraint (physical and/or chemical); and reduction in adverse events, such as falls. The review is registered with PROSPERO, Review Registration Number: PROPSERO 2018 CRD420181C0431.

### Review criteria

The review of studies on PCC in healthcare services for people with dementia was undertaken in accordance with the review protocol [27]. We included randomized and cluster-randomized controlled clinical trials, and quasi-experimental studies published in English, which evaluated the effectiveness of providing PCC at the organizational level for people with dementia in formal healthcare services, according to the VIPS requirements [24], compared to care that was routinely undertaken in healthcare services, i.e. non-PCC. Healthcare settings included long-term care homes, hospitals and community-based services. Study participants included people diagnosed with dementia according to the Diagnostic and Statistical Manual of Mental Disorders (DSM-5) [28] and/or the International Classification of Diseases (ICD) [29].

We excluded studies that were: not specific to dementia; focused on dementia caregiver support and/or burden; and directed at early detection of dementia; and studies which tested targeted interventions for dementia, including psychosocial approaches, that were not identified as PCC.

#### Search strategy and inclusion/exclusion of studies

Author LC executed the search strategy, assisted by JSP and SL. Bi-monthly searches were conducted in ALOIS [30] the Cochrane Dementia and Cognitive Improvement Group Specialised Register, from 1 June 2016 to 1 June 2018. The search terms included: Dementia, Delirium, Amnestic, Cognitive Disorders, dement*, alzheimer*, organic brain disease or organic brain syndrome; cerebral*; AND, activity, activities, psychosocial, non-pharmacological, individually-tailor*, personally-tailor*, individual or individuals or individually-cent*, meaning* or meaningful*, engagement or engaging, occupational*, personhood, person-centred, patient-centred care; AND; randomized controlled trial, controlled clinical trial, randomly, placebo, randomized, randomised, double-blind* or single-blind* RCT or CRCT. The following databases were accessed: MEDLINE (via the Ovid SP platform), EMBASE, CINAHL, PsycINFO, and LILACS; Trial registers: meta Register of Controlled Trials, Umin Japan Trial Register, WHO Clinical Trials Registry Platform portal, ClinicalTrials.gov, ISRCTN, Chinese Clinical Trial Register, German Clinical Trials Register, Iranian Registry of Clinical Trials, Netherlands National Trials Register, and Central Register of Controlled Trials (CENTRAL) in the Cochrane Library; Grey literature sources: ISI Web of Knowledge Conference Proceedings; Index to Theses, and Australasian Digital Theses; NHS Economic Evaluation Database (NHS EED). In addition, LC, JSP and SL undertook snowballing of the reference list of all studies that were suitable for review. At this stage of the search no restrictions were placed on articles accessed in relation to their methodological quality. The search that was used for the retrieval of reports of trials from MEDLINE (via the Ovid SP platform) can be found in S1 Fig.

The search results for individual databases were combined and duplicate records were removed. LC and JSP independently reviewed the titles and abstracts of each study to determine their eligibility for inclusion in the review, based on the review aims and criteria for inclusion. LC and JSP discussed any disagreements about study eligibility after reviewing the full published articles which met the review criteria, and referred any that were unresolved to reviewer SL.

#### Methodologic quality assessment

Reviewers SL and AYW independently assessed the quality of studies using criteria outlined in the Cochrane Handbook for Systematic Reviews of Interventions [31] using the GRADE criteria [32]. This set of criteria includes evidence of associations between overestimate of effect and high risk of study bias, such as sequence generation, allocation concealment, blinding, incomplete outcome data and selective reporting. On review of the included studies, there was a conflict of interest for two of them which were authored by reviewers LC and JSP [33,34]. Consideration of these two studies for inclusion in the review was determined by an external reviewer (EB), who used the same Cochrane criteria and GRADE to independently review these two studies. Due process was followed in dealing with review author conflict of interest [32].

#### Data extraction

LC and JSP independently extracted data from each included study using the Template for Intervention Description and Replication (TIDieR) checklist [35]. Information recorded included country of origin, publication date, trial registration number and ethical approval details, number of participants receiving each intervention, gender, age, type of dementia diagnosis, study setting, description of control and PCC intervention components, total duration of the PCC intervention, follow-up period, outcome measurements targeted and results for experimental and control study groups. The Templates were cross-checked by authors LC, JSP, SL and AYW.

The corresponding authors of five included studies [33,34,36,37,38] were contacted by author SL to obtain further information on statistical tests and test results for primary outcomes of interest. Further information was provided on all statistical tests used, as well as raw group means and standard deviations (SD) for four of the studies [33,34,36,38]. For one study [37] where these data were not available, the following procedure was followed: where there were missing measures of variance for continuous data, but an exact standard error (SE) and/or Confidence Intervals (CI) available for group means, and either P value or t value available for differences in mean, SL, ZL and AYW calculated the SDs using generic inverse variance calculator according to the rules described in the Cochrane Handbook for Systemic reviews of Interventions [31]. These results were included in the data extraction tables.

#### Review of person-centred care interventions across studies

The TIDieR checklist [35] was used by authors LC and JSP to list the components of the PCC interventions for the 12 studies reviewed. The intervention components were reviewed against the four elements of the VIPS framework [24] and the PCECAT tool [26] for implementation of PCC at the organisational level.

#### Statistical analysis

Analysis were undertaken by ZL, AYW and SL for all primary and secondary outcome data, measuring standardized mean difference (SMD) on an intention-to-treat basis for all study participants. The denominator for each outcome was the number randomized, minus any participants whose outcomes were known to be missing, with the cluster constituting the unit of analysis. The reported methods accounting for cluster randomization in each study were accepted as appropriate, however, if methods were not reported and unavailable, the data were re-analyzed following the Cochrane Handbook guidelines [31].

Statistical heterogeneity was assessed using the I^2^ method and Chi^2^ P value [39]. Heterogeneity was regarded as substantial if an I^2^ was greater than 75% and accompanied by a statistically significant Chi^2^ statistic as evidence. The results were presented as the average treatment effect with 95% confidence intervals, and the estimates of Tau^2^ and I^2^. SL, AYW and ZL undertook additional analysis using a random-effects model with restricted maximum likelihood estimation, for first/last time point data within 12-months in R [40]. Data were further analyzed applying unit of analysis errors according to Effective Practice and Organisation of Care (EPOC) [41] guidelines and using absolute risk differences [42].

Sensitivity analyses were then conducted for risk of bias [32] by each outcome, in one or more of the domains of randomization (i.e. implied as randomized with no further details available), allocation concealment, blinding and outcome reporting for the meta-analysis of the primary outcome. If the exclusion of trials at high risk of bias did not substantially alter the direction of effect or the precision of the effect estimates, then data from these trials were included in the analysis [31].

## Results

### Literature search and study characteristics

**Fig 1:**
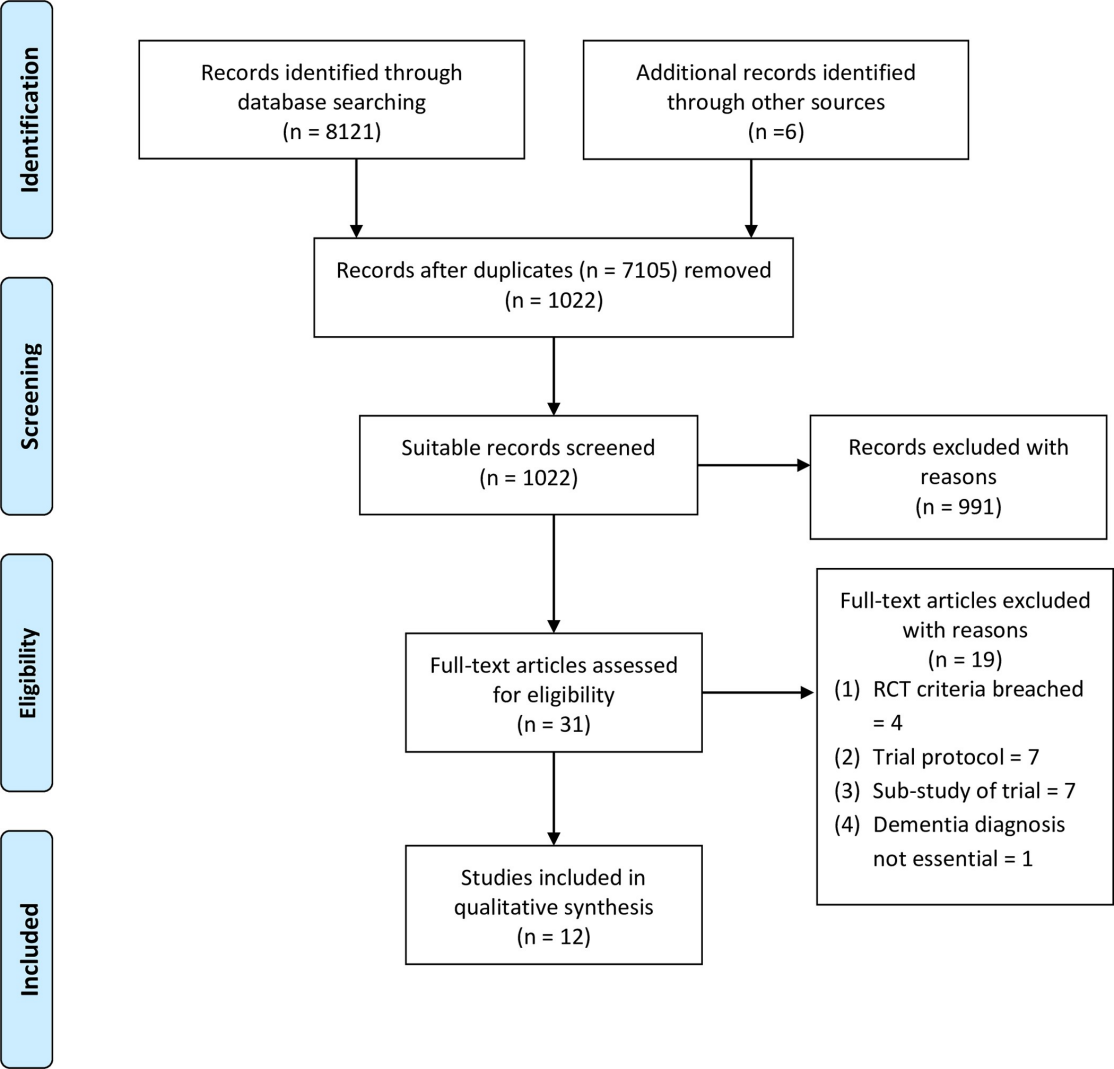
PRISMA [43] search strategy.

The search yielded a total of 8127 study titles and abstracts from ALOIS [30], 1022 remained after the removal of 7105 duplicates and were subjected to title and abstract screening, and 991 of the studies which failed to meet the review inclusion criteria [27] (i.e. experimental studies, focusing on people with dementia, organization-level PCC interventions) were removed. Only 31 of the remaining studies met the review inclusion criteria on reading the study abstracts. Full-text versions of these 31 studies were reviewed against the predefined eligibility criteria [27]. Nineteen of the 31 studies were excluded from full review as four breached the RCT criteria through planned control group exposure to PCC, seven included PCC trial protocols, seven were sub-studies of PCC trials, and one study included some participants without dementia. The full review yielded 12 studies fulfilling all review criteria (N = 3402 study participants) (Fig 1). Of these 12 studies two were conducted in Australia, one in Germany, two in the Netherlands, one in Scandinavia, four in the UK and two in the USA.

A breakdown of the studies eligible for inclusion in the review included 11 cluster-randomized controlled trials (CRCT) [15,33,34,36,38,44–49] and one quasi-experimental study [37]. All 12 studies compared PCC with usual care, i.e. non-PCC, using multi-modal PCC approaches at the organizational level (Table 1). While there were some differences in the way that PCC was implemented, all of the 12 studies adhered to the four VIPS [24] elements and systems requirements as follows:

**Table 1:** Characteristics of included studies.

Author Year Country	Methods	Study setting	Participant Characteristics	Intervention Characteristics	Primary outcome	Secondary outcomes	Risk of bias rating *	
Brooker et al. 2011 UK	Cluster randomised controlled trial, with four measurement points: baseline, 6, 12 and 18 months	10 extra care housing schemes	293 residents Age: 81.50 ± 8.05 Gender: 221 women, 72 men	Person-centred Enriched Opportunities Programme (EOP) was compared with Care as Usual (control) Duration of EOP 12 months	Quality of Life (QOL-AD)^1^	Mood—Depression (GDS)^2^, Mood- Well-being (DCM-WIB)^3^, Social integration and support (DSSI)^4^, Occupation/activity engagement (chart review), Transfer to hospital/ high level care (chart review)	Selection bias	Low
							Performance bias	High
							Detection bias	High
							Attrition bias	Low
							Reporting bias	Low
Chenoweth et al. 2009 Australia	Cluster randomised controlled trial, with three measurement points: baseline, 4 and 8 months	15 accredited aged care units within 12 residential aged care homes	289 residents Age: Mean age of participants for each arm: Dementia care mapping: 83 ± 7.6 Person-centred care: 84 ± 6.4 Usual care: 85 ± 6.6 Gender: 224 women, 65 men	Two PCC approaches (PCC and DCM) were compared with each other and with Care as Usual (control). Duration of PCC and DCM interventions 8 months	Agitated behaviour (CMAI)^5^	Neuropsychiatric symptoms (NPI-NH)^6^, Quality of life (QUALID)^7^, Quality of care interactions (QUIS)^8^, Well-being (DCM-WIB)^3^, Psychotropic drug use (chart review), Adverse events and associated medical consults and hospitalisation (chart review), Care manager support for PCC champions and DCM trained staff (survey), Cost benefit of PCC and DCM implementation (economic analysis)	Selection bias	Low
							Performance bias	High
							Detection bias	Low
							Attrition bias	Low
							Reporting bias	Low
Chenoweth et al. 2014 Australia	Cluster randomised controlled trial, with three measurement points: baseline, 4 and 8 months	38 aged/dementia care units within 38 residential aged care homes	601 residents Age: Mean age of participants for each arm: Person-centred care: 84 ± 8 Person-centred environment: 84 ± 8 Person-centred care + person-centred environment: 84 ± 7 Usual care: 86 ± 7 Gender: 481 women, 120 men	Three person-centred service approaches (PCC, PCE, PCC+PCE) were compared with each other and with Care as Usual (control). Intervention Duration for PCC 8 months, for PCE 6 months and for PCC+PCE 6–8 months	Agitated behaviour (CMAI)^5^, Quality of life (DEMQOL)^9^, Emotional responses in care (ERIC)^10^	Quality of care interactions (QUIS)^8^, Mood depression (CSDD)^11^, Psychotropic drug use (chart review), Adverse events and associated medical consults and hospitalisation (chart review), Cost benefit of PCC, PCE and PCC+PCE implementation (economic analysis)	Selection bias	Low
							Performance bias	Low
							Detection bias	Low
							Attrition bias	Low
							Reporting bias	Low
Cohen-Mansfield et al. 2012 USA	Randomised placebo-controlled clinical trial, with two measurement points: baseline, 2 and 3 weeks	9 nursing homes	125 residents, > = 60 years old Age: Mean age of residents at baseline: All residents: 85.7 ± 8.89 Intervention group: 85.9 ± 8.62 Control group: 85.3 ± 9.62 Gender: 93 women, 32 men	Person-centred, non-pharmacological management of agitation TREA (Treatment Routes for Exploring Agitation) was compared to Care as Usual plus in-service staff education (control). Duration of TREA 2 weeks	Agitation (ABMI)^12^, Pleasure, interest and mood (LMBS)^13^	Anti-depressant and anti-anxiety medication use (chart review)	Selection bias	Low
							Performance bias	High
							Detection bias	Low
							Attrition bias	Low
							Reporting bias	Low
Dichter et al. 2015 Germany	Pragmatic quasi-experimental trial, with three measurement points: baseline, 6 and 18 months	9 nursing homes	Age: Dementia care mapping with prior experience group: 82 ± 6.8 Dementia care mapping with no prior experience group: 84.1 ± 6.3 Usual care group: 82.6 ± 9.2 Gender: 128 women, 26 men at baseline 40 new residents were recruited during the course of the study	Person-centred intervention DCM was compared for staff with prior DCM experience and staff with no prior DCM experience and with Care as Usual (control) Duration of DCM 12–18 months	Quality of Life (QOL-AD^1^ proxy) and (QUALIDEM)^7^	Neuropsychiatric symptoms (NPI-NH)^6^	Selection bias	High
							Performance bias	High
							Detection bias	High
							Attrition bias	Low
							Reporting bias	Low
Finnema et al. 2005 Netherlands	Cluster randomised control trial, with three measurement points: baseline, 3 and 7 months	16 nursing home units	194 residents Age: Mean age of residents at baseline: Intervention group: 83.8 ± 5.3 Control group: 83.6 ± 5.8 Gender: Not reported	Person-centred care using emotion-oriented approaches was compared with Care as Usual (control) Intervention Dose 100% Intervention Duration of PCC 7 months	Adaptation-coping (BOP [Beoordelingsschaal voor Oudere Patie¨nten])^14^	Mood-Depression (CSDD)^11^ Morale (PGCMS)^15^ Agitation (CMAI)^5^, Staff Self-rated Health (GHQ-28)^16^, Staff work satisfaction and sense of competence (Dutch Work Satisfaction Scale)^17^	Selection bias	Low
							Performance bias	High
							Detection bias	Unclear
							Attrition bias	Low
							Reporting bias	Low
Fossey et al. 2006 U.K.	Cluster randomised controlled trial, with three measurement points: baseline, 3 and 12 months	12 specialist nursing homes for people with dementia	349 residents Age: Median age and range of residents at baseline: Intervention group: 82 (60–98) Control group: 82 (53–101) Gender: 130 women, 219 men	Person-centred non-pharmacological management of neuropsychiatric symptoms was compared to Care as Usual (control). Duration of PCC 10 months	Neuroleptic prescribing/use and dose (chart review) Other psychotropic drug prescribing and use (chart review)	Agitation (CMAI)^5^, Mood- Well-being (DCM-WIB)^3^, Incidents of irritable and aggressive behaviour (chart review) Adverse events (incl. falls) (chart review)	Selection bias	Low
							Performance bias	Unclear
							Detection bias	Unclear
							Attrition bias	Low
							Reporting bias	Low
Goldberg et al. 2013 U.K.	Cluster randomised controlled trial, with three measurement points: baseline, at discharge and 3 months	Acute general hospital	600 patients aged over 65 admitted for acute medical care Age: Median age and range of residents at baseline: Intervention group: 85 (80–88) Control/standard care group: 85 (80–89) Gender: 312 women, 288 men	Person-centred approach to delirium prevention and management in people with dementia were compared to Care as Usual services (control). Duration of PCC 90 days (median 11 days/patient)	Number of days spent at home after hospitalisation, Number of days spent in hospital, Mortality rate, Re-admission to hospital, In-patient rehabilitation or intermediate care New placements in a care home (chart and hospital record reviews)	Mood-well-being (DCM-WIB)^3^, Quality of life (DEMQOL proxy)^9^ and EuroQolEQ-5D)^18^ Disability (Short London Handicap Scale)^19^, function (Barthel Index)^20^, Neuropsychiatric symptoms (NPI)^21^, Carer strain (CSI)^22^, Carer self-reported health (GHQ-12)^23^, Carer satisfaction	Selection bias	Low
							Performance bias	High
							Detection bias	Low
							Attrition bias	Low
							Reporting bias	Low
Lawton et al. 1998 USA	Cluster randomised control trial, with three measurement points: baseline, 6 and 12 months	Two equivalent/matched special care nursing home units	182 residents Age: not reported Gender: not reported	Person-centred care using the Stimulation-Retreat model, compared to Care as Usual (control). Duration of PCC 12 months	Affective state/Pleasure (MOSES)^24^ and (AARS)^25^, Mood- Depression (MDS)^26^, Social Quality, Time Use, Sociability (MDS)^26^ and (MOSES)^24^, Gazing with Interest, Length of emotion display in activities (AARS)^26^ and (MOSES)^25^	Aggression/irritability (BEHAVE-AD)^27^, Agitation (CMAI)^5^ and (BRS)^28^, Repetitive behaviour (MDS)^26^, Functional health (PSMS)^29^, Cognition (GDS)^2^	Selection bias	High
							Performance bias	High
							Detection bias	High
							Attrition bias	Low
							Reporting bias	Low
Li et al. 2015 USA	Cluster randomised controlled trial, nested within a larger study, with two measurement points: baseline and 1 month	Two secure dementia care units	26 residents ≥ 65 years old Age: Mean age for all residents: 86.45 ± 6.90 Intervention group: 85.67 ± 5.16 Control group: 88.66 ± 5.16 Gender: Not reported at baseline	Person-centred care, compared with Care as Usual (control). Duration of PCC 4 weeks	Total hours and % of sleep in 24 hours (Actiwatch)^30^ Number of awakenings from sleep in 24 Hrs (Actiwatch)^30^ Daytime physical and social activity (Actiwatch)^30^ Daylight exposure (Light sensor)	Social and physical engagement (DCM-WIB)^3^	Selection bias	High
							Performance bias	High
							Detection bias	High
							Attrition bias	Low
							Reporting bias	Low
Rokstad et al. 2013 Norway	Cluster-randomized controlled trial with two measurement points: baseline and 10 months	15 nursing homes with a total of 40 units	624 residents Age: Mean age for all residents:85.7 ± 8.3 Dementia care mapping group: 85.1 ± 8.7 VIPS practice model group: 85.1 ± 8.7 Control group: 87.0 ± 8.3 Gender: 448 women, 176 men	Two person-centred care approaches (DCM and VPM) were compared with each other and with Care as Usual (control) Duration of DCM and VPM 10 months	Agitation (BARS)^31^	Neuropsychiatric symptoms (NPI)^21^, Mood Depression (CSDD)^11^, Quality of life (QUALID)^7^ Activities of daily living (PSMS)^29^ General health (GMHRS-modified)^32^	Selection bias	Low
							Performance bias	High
							Detection bias	Low
							Attrition bias	Low
							Reporting bias	Low
Van de Ven et al. 2013 Netherlands	Cluster randomised controlled trial, with three measurement points: baseline, 4 and 8 months	11 nursing care homes and 34 units	192 residents with dementia Age: Mean age of residents at baseline: Intervention group: 84.6 ± 6.1 Control group: 83.5 ± 6.6 Gender: 143 women 49 men	The DCM approach to Person-Centred Care was compared with Care as Usual (control) Duration of DCM 8 months	Primary outcome: Agitation (CMAI)^5^	Neuropsychiatric symptoms (NPI-NH)^6^, Quality of life (Qualidem)^33^ and (EuroQol 5D)^18^, Staff health and stress (GHQ-12)^23^, Staff Job satisfaction (MJSS-HC)^34^	Selection bias	Low
							Performance bias	High
							Detection bias	Low
							Attrition bias	Low
							Reporting bias	Low

*Risk of bias rating adopted from the Cochrane Collaboration’s tool for assessing risk of bias

References for study measures 1–34 can be found in S1 Table.

Across all 12 studies, there was an emphasis on valuing the service user and the service staff. Executive and management staff established systems to support the adoption of PCC among all levels of staff, and they empowered the trained PCC champions/coaches to assist direct service staff in reconceptualizing care and therapy practices. All 12 studies focused on developing direct care staff and care manager knowledge, attitudes and skills through staff training in PCC, complemented by PCC skills modelling and supervision by PCC champions/coaches, and managerial leadership for PCC at a systems-level. One hospital-based study [38] included all members of the healthcare team in learning how to apply PCC in recognizing, preventing and managing delirium and agitation more therapeutically. Additionally, all 12 studies educated, trained and supervised direct care staff to undertake person-centered care planning in consultation with the service user and/or their family members, which formed the basis for providing individualized care. The same processes occurred for planning and delivering social, therapy and activity programs targeted to individual strengths, needs and preferences, in which family members were encouraged to participate. This emphasis on provision of individualized care and therapy programs, aimed to acknowledge and support unique service user strengths and vulnerabilities across a wide range of needs. Person-centered, non-pharmacological approaches to reducing neuropsychiatric symptoms, such as agitation, was a motif of all 12 studies.

This was enabled through targeted staff education and supervision, champion/coach role modelling, casework discussions among the team, and managerial leadership, which aimed to change staff attitudes to ways of caring for people with dementia. In achieving this goal, direct care staff were encouraged to seek out information on the individual life stories, personalities and achievements, and to employ this knowledge in delivering therapeutic care. Staff were also encouraged to draw on this knowledge in developing closer relationships with the service user and their family members, and to facilitate a more engaged social environment. This also occurred through provision of a greater variety of activities available to meet needs and abilities of service users. Four studies [15,34,38,49] adapted the care environment using person-centered environmental principles, making it less restrictive, more comfortable, recognizable, interesting and interactive, and helping to orient the service user to the current situation while supporting their capabilities.

Comparison groups in all 12 studies provided usual (non-person-centered) care, social and recreation activity programs in other care units, wards or services without any planned exposure to person-centered models and systems. Follow-up assessment ranged from 3 weeks to 3 months [36,44,45], 4 to 8 months [33,34,38,48] and 10 to 18 months [15,37,46, 47,49].

The mean participant ages were 81–86.45 years and 65% were female, and all participants had a diagnosis of dementia and presence of neuropsychiatric symptoms that caregivers found troublesome. Participant attrition ranged from 20–30% in the 10 studies undertaken in long-term care homes. Most of the participants had more than one comorbidity and all had some functional impairment associated with older age and/or dementia.

Table 1 summarizes the participant, site and study characteristics, study intervention and measurement, and the risk of bias ratings of all 12 studies.

### Primary participant outcomes

**Fig 2:**
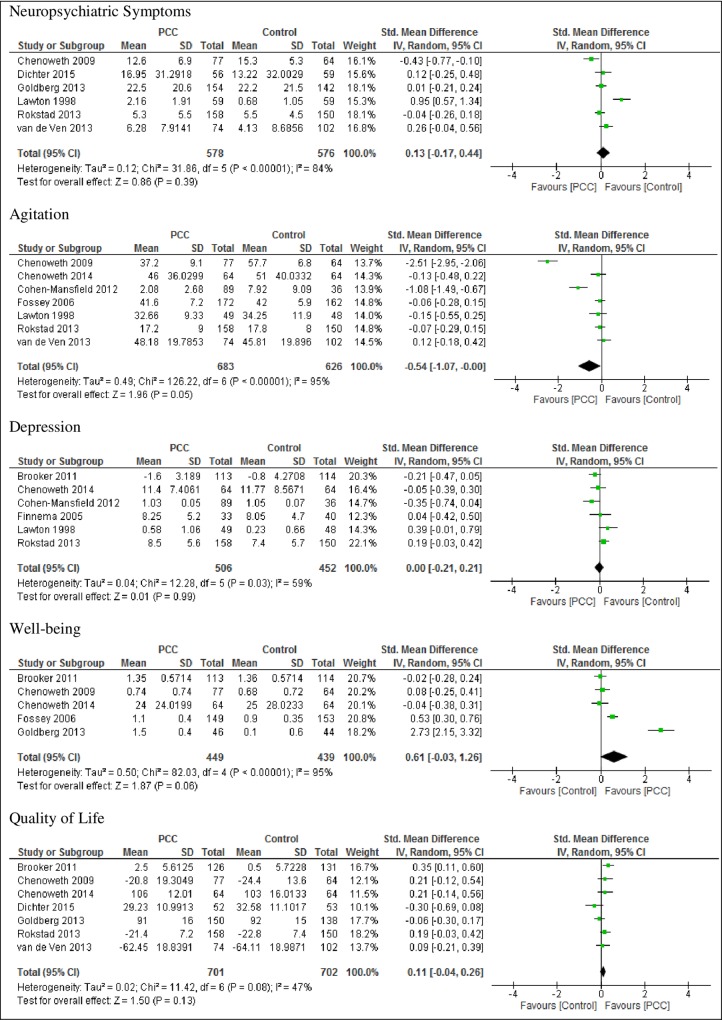
Forest plots of primary outcomes from all studies.

**Fig 3:**
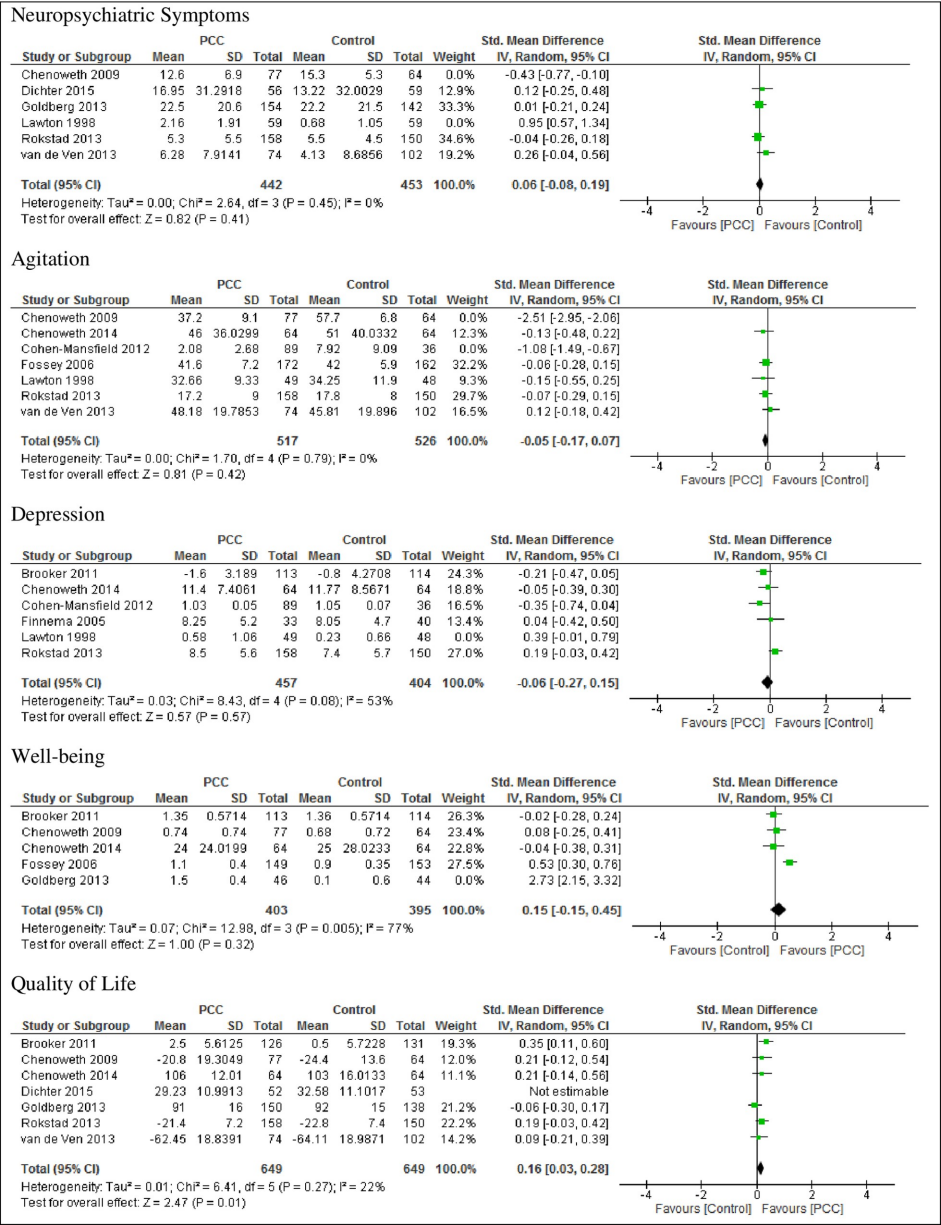
Forest plots of all studies with low heterogeneity.

The results of analyses of pooled data for the primary participant outcomes from studies measuring these outcomes are presented in Fig 2, followed by results of the sensitivity analysis in studies with low statistical heterogeneity in Fig 3.

#### Neuropsychiatric symptoms

Six studies [33, 37,38,46,47,48] reported a non-significant reduction in the standardized mean score of neuropsychiatric symptoms with person-centered care compared with usual care (SMD 0.13, 95% CI -0.21 to 0.49; studies = 6; I^2^ = 88%) (Fig 2). Sensitivity analysis was not conducted since only two of the studies had low risk of bias [33,47] (Table 1). In four of the studies with low heterogeneity [37,38,47,48] there was a non-significant reduction in neuropsychiatric symptoms (SMD 0.06, 95% CI -0.08 to 0.19; studies = 4; participants = 895; I^2^ = 0%) as shown in Fig 3.

#### Agitation

Eight studies [15,33,34,36,44,46,47,48] produced a non-significant reduction in agitation (SMD -0.54, 95% CI -1.23 to 0.15; studies = 8; I^2^ = 97%). Sensitivity analysis of five studies with low risk of bias [15,33,34,36,47] showed an increased overall effect for the person-centered care group (p < 0.00001, SMD 0.38, 95% CI -0.50 to -0.25). Analysis of five studies with low heterogeneity [15,34,46,47,48], however, showed no significant difference in agitation between the person-centered care group and the control group; SMD -0.05 (95% CI -0.17 to 0.07; studies 5; participants = 1043; I^2^ = 0%).

#### Depression

Six studies [34,36,44,46,47,49] produced no difference in the standardized mean depression score (SMD 0.00, 95% CI -0.21 to 0.21; studies = 6; I^2^ = 59%). Sensitivity analysis of four studies with low risk of bias [34, 36,44,47] similarly showed no significant differences in mean depression scores for person-centred care or usual care (p = 0.87, SMD 0.01 and 95% CI -0.13 to 0.16). Five of the studies with low heterogeneity [34,44,46,47,49] showed no change in depression with person-centered care or usual care (SMD -0.06 and 95% CI-0.27 to 0.15, studies = 5; participants = 861; I^2^ = 53%).

#### Well-being

Six studies [15,33,43,38,45,49] showed a non-significant increase in the standardized mean well-being score in person-centered care compared to usual care (SMD 0.64, 95% CI -0.37 to 1.65; studies = 6; I^2^ = 98%). After excluding two studies with high risk of bias [38,45] the overall mean score in the person-centered care group remained significantly higher than in the usual care group (p = 0.002, SMD 0.25, 95% CI 0.09 to 0.40). Four of the studies with low heterogeneity [15,33,34,49] however, produced a non-significant improvement in well-being with person-centered care (SMD 0.15, 95% CI -0.15 to 0.45; studies = 4; participants = 798; I^2^ = 77%).

#### Quality of life

Seven studies [33,34,37,38,47,48,49] produced a non-significant improvement in standardized mean quality of life scores with person-centered care, compared with usual care (SMD 0.11 and 95% CI -0.04 to 0.26; studies = 7; I^2^ = 47%). Analysis of three studies with low risk of bias (33,34,47] showed a significant effect in the quality of life score in the person-centered care group (p = 0.02, SMD 0.2, 95% CI 0.04 to 0.36). Six of the studies with low heterogeneity [33,34,38,47,48,49] showed a significant improvement in quality of life with person-centered care (SMD 0.16 and 95% CI 0.03 to 0.29; studies = 6; participants = 1298; I^2^ = 29%).

### Secondary participant outcomes

Data for secondary outcomes, function in activities of living, physical and chemical restraint and adverse events, were neither sufficient nor sufficiently similar to undertake an analysis. Nevertheless, a review of the reported results of individual trials indicated a mixed trend towards improvement in some activities of living, mainly physical function [33,36,44,45,46] and engagement in leisure/social activities [33,34,45,49] (studies = 7; participants = 1298; p = 0.01 to 0.58), and also a trend towards a reduction in the use of physical restraint [33,34] (studies = 2; participants = 890; p = 0.02 to 0.006). There was variable success in reducing use of chemical restraint with person-centered care [33,48] (studies = 2; participants = 915; p = 0.08 to 0.66), and in preventing adverse events such as falls [33,34,36,48] (studies = 4; participants = 1109; p = 0.03 to 0.27).

## Discussion

The review identified 12 studies that met the inclusion criteria for assessing the effectiveness of PCC delivered at the organizational level for people living with dementia as recommended in the VIPS guidelines [23] and the PCECAT instrument [26]. While the results showed a significant effect for increased quality of life, and a non-significant improvement in neuropsychiatric symptoms and well-being, there was no evidence that demonstrated a reduction in agitation and depression. Apart from demonstrating no effect for agitation, these findings concur with the results of three systematic reviews of PCC for people living with dementia [14,50,51]. Similar with previous findings [51,52], delivery of PCC at the organizational level did not have a positive impact on level or rates of depression in people living with dementia.

In all likelihood, the mixed trial results for reducing agitation specifically, one of the most common types of neuropsychiatric symptoms experienced by people with dementia, may reflect the intention of most studies to obtain these data in relation to the person’s behaviour in general, rather than in relation to specific care events, such as during personal care when an agitation response is more likely to occur. Another possible reason for the mixed results for agitation is the complexity of an organizational-wide implementation of the PCC approach. Implementation of PCC is dependent upon a number of factors that are not always measured in PCC trials, for example, organizational culture, staffing levels, staff skill-sets and mix, and the physical and psycho-social care environment.

There can be limited confidence in the findings of this review, given the moderate to high methodological quality of the 12 included studies in relation to the clear reporting of the study designs and methods. A majority of the studies had low risk of bias overall, with the exception of performance bias common in CRCTs and quasi-experimental studies. All 12 studies included participants of similar ages and clinical characteristics, including a valid diagnosis of dementia, and exhibiting neuropsychiatric symptoms which caregiving staff found troublesome. As expected in this vulnerable population, there was a 25–30% participant loss-to-follow-up in studies conducted in the long-term care setting, mainly due to the death of participants. The variation in time to follow-up in the different studies, ranging from 6–18 months in the long-term care setting and two to 3 weeks in the hospital setting, is explained by the focus of these different settings and thus, the longer predicted length of stay in the long-term care setting compared with the hospital setting. Being able to achieve improved quality of life in people with advanced dementia through the delivery of PCC in both of these settings has important clinical and policy implications.

Another cause for confidence in the review findings is that all 12 studies obtained baseline and follow-up outcome data using validated measures for the primary outcomes and for most of the secondary outcomes. Many of the studies employed the same validated outcome measures (refer Table 1), which enabled robust statistical analyses of the combined results.

While all studies showed improvements in at least one primary outcome using multi-modal implementation of PCC as outlined in the VIPS guidelines [24], the implementation procedures were not standardized across studies. All of the studies did adhere, however, to the principle of aiming to support personhood through the provision of individualized care, therapy and activity programs by all direct caregivers and therapy staff, as well as adhering to the principle of requiring all staff to interpersonally communicate and engage with the person in ways that encouraged their self-determination, self-respect, dignity and well-being.

Another common feature of all studies was the operation of PCC at the organizational-level, as recommended in the VIPS framework [24]. Organisational-level interventions included a combination of: staff education, training, guidance and exposure to role modelling in how to understand and interpret verbal and non-verbal communication from the perspective of the person with dementia; and providing the person with interesting, purposeful, and meaningful things to do in their daily life, in consultation with the person, their family and caregivers. Person-centered care and leisure/social activities all included approaches that were meaningful for the person, such as communicating with them about their memories of family, friends, places and events. When the whole care team employed this approach, it was found to increase the person’s participation in care and leisure/social activities, which in most studies also improved psychosocial and functional outcomes, albeit non-significantly.

These outcomes support Kitwood’s [7] theoretical assumption that positive interpersonal relationships and enriched care environments can prevent the disabling effects of dementia and promote a sense of well-being for the person [6,8,9]. A recent study evaluating an organisation-wide PCC model in Norwegian aged care homes found a positive relationship between a person-centered organisation, care staff work practices and organizational systems established to support the model [52].

### Limitations

A limitation of the review was the difference in measurement points across studies, varying from three weeks in the hospital setting at follow-up to 18 months follow-up in the long-term care and community-based settings. Variance in data collection points occurred because of the different functions of the study settings and the aims of the different PCC intervention programs, as well as the differences in anticipated participant lengths-of-stay in these very different care settings. The decision was, therefore, made to undertake an analysis of all primary outcomes with data obtained within 12-months after baseline. This limitation needs to be considered when analysing the effects of PCC in studies within different contexts and follow-up periods.

As identified, depending on the study context and anticipated participant loss to follow-up of between 25–35%, the included studies had variable duration of the PCC intervention as follows: 3–4 weeks [36,45]; 3 months [44], 7–8 months [33,34,38,48], 10–12 months [15,46,47], and 18 months [37,49]. However, other than in the two studies that had very short follow-up assessment periods [36,45] where there was no or minimal participant dropout, the differences in the duration of PCC in the remaining 10 studies did not appear to have a measurable effect on the primary outcomes assessed.

Another limitation of the analyses is that while 10 of the12 included studies used more than one measure to assess some of the primary and secondary outcomes, the results of only one of the most frequently used primary outcome measures were analysed, e.g. pooled agitation results were analysed for the seven studies which reported the Cohen-Mansfield Agitation Inventory scores [53] (Refer to Table 1 for all outcome measures). The results may have changed if the primary outcome results had included the pooled data of all the different outcome measures used to assess the same constructs, e.g. where more than one outcome measure was used in any one study to assess quality of life.

A possible limitation of the review was the inclusion of studies with moderate to high risk of bias [37,46, 49]. These studies had a high risk of bias in three or more areas, including for neuropsychiatric symptoms [37,46], agitation [46], depression [47,49] and quality of life [37]. Although these studies’ results were included in the initial analysis (Fig 2), they were excluded when undertaking additional analyses of low bias studies, which produced more favourable results in the person-centred care group for agitation, well-being and quality of life.

Analysis of secondary outcomes was not possible because there were insufficient data using comparable instruments. In the case of activities of living, for example, it was not possible to pool the data in studies using the DCM [54] activities of living codes and data obtained with other validated measures. As well, there was statistical heterogeneity of study results and an insufficient number of studies to undertake analyses of secondary outcomes.

### Implications for research

This review has highlighted a number of issues which should be considered when designing future research on the organization-wide implementation of PCC. It would be useful to standardize methods of education, training and supervision of PCC and person-centred recreation, social and therapy programs, in order to compare outcomes for different study populations and across various care settings, and in people with mild, moderate and severe dementia. Minimally, the specific approaches to PCC education, training and supervision that have been employed should be reported, thus enabling comparison across studies. As well, it will be important to determine the required minimum number of hours of person-centred education, training and supervision required, the optimal dose and duration of PCC support required, and the dose of the individual components of PCC operating at the organisational level that is required, to produce positive outcomes for the person living with dementia.

As different contexts of care (acute, supported care housing, long-term care) will determine the potential dose and duration of PCC interventions, it will be important to distinguish the immediate and longer-term outcomes for people living with dementia in these different care situations (or contexts). Further research is needed to evaluate PCC outcomes at critical points in the person’s care trajectory, such as when a significant change in health status occurs. Additionally, further assessment of the delivery of PCC in different locations (hospital and community-based settings) and assessment of care provision by different types of staff, carers and family, would provide valuable information to understand how organizations can best support and deliver the PCC model across the whole organization. This is particularly important when considering how best to provide supportive services and care for people with terminal dementia in ways that are supportive of their end-of-life needs.

Since there is considerable overlap between neurological symptoms, mood and quality of life in people with dementia, and multiple factors involved in their expression, it is important when assessing these outcomes to consider the inter-relationships between the person, their caregivers and features of the care environment. It may be useful, therefore, to measure a number of dimensions associated with the outcomes of interest including physiological, psychological, spiritual, social, and personal preferences, and to recognize that various dimensions may hold different salience for individuals living with dementia. Consequently, novel and innovative approaches are required to evaluate the benefits of particular organizational systems and care practices for the individual. None of the included studies undertook a comprehensive cost-effectiveness analysis of the models of PCC implemented, therefore, future research is urgently required to assess the efficiencies as well as the effectiveness of quality aged care systems.

### Conclusions

This systematic review of organizational-level implementation of PCC suggests that where PCC operates at the organizational level, with the full support of organizational leaders, it can increase quality of life in people living with dementia, and it can potentially improve their well-being and reduce neuropsychiatric symptoms. While dementia is a progressive, incurable illness, improving the quality of life for people who experience it is of clinical significance. Maintaining dignity and personhood in the face of this illness is consistent with the humanistic values underpinning quality health care.

## Supporting information

S1 Table Included studies measurement references. (DOCX)

S2 Table PRISMA checklist plos 1. (DOC)

S1 Fig MEDLINE search strategy. (DOCX)
