# Supplementary material for: Effects of person-centered care at the organisational-level for people with dementia. A systematic review
Source: PLoS One. 2019 Feb 22;14(2):e0212686. doi: 10.1371/journal.pone.0212686 (PMC6386385; doi:10.1371/journal.pone.0212686)
Supplement: S1 Table — (DOCX) [file pone.0212686.s001.docx]

**S1 Table.** **Included Studies Measurement References**

^1^ Logsdon R, Gibbons L, Teri L. Quality of Life in Alzheimer’s Disease: Patient and Caregiver Report (QOL-AD). J Ment Health Aging. 1998; 5: 21–32.

^2^ Sheikh, J., & Yesavage, J. Geriatric depression scale: recent evidence and development of a shorter version. In: Brink T.L, editor, Clinical gerontology: a guide to assessment and intervention. New York: Haworth; 1986. pp. 165–173.

^3^ Bradford Dementia Group. DCM 8 user's manual. Bradford: University of Bradford; 2005.

^4^ Koenig HG, Westlund RE, George LK, Hughes DC, Blazer DG, Hybels C. Abbreviating the Duke Social Support Index for use in chronically ill elderly individuals. Psychosomatics. 1993; 34: 61–69.

^5^ Cohen-Mansfield J. Measurement of inappropriate behaviour associated with dementia. J Gerontol Nurs. 1999; 25: 42–51.

^6^ Wood S, Cummings JL, Hsu M-A, Barclay T, Wheatley MV, Yarema KT, et al. The use of the Neuropsychiatric Inventory in nursing home residents, characterization and measurement. Am J Geriatr Psychiatry. 2000; 8: 75-83.

^7^ Weiner M, Martin-Cook K, Saine K, Foster B, Fontaine C. The quality of life in late-stage dementia (QUALID) scale. J Am Med Disord Assoc. 2000; 1**:** 114–16.

^8^ Dean R, Proudfoot R, Lindesay J. Quality of interactions schedule (QUIS). Int J Geriatr Psychiatry. 1993; 8: 819–26.

^9^ Smith SC, Lamping DL, Banerjee S., Harwood R, Foley B, Smith P, et al. Measurement of health-related quality of life for people with dementia: development of a new instrument (DEMQOL) and an evaluation of current methodology. Health Technol Assess. 2005; 9(10): 1–93.

^10^ Fleming, R. Emotional Responses in Care Assessment (ERiC). Sydney, Australia: The Hammond Care Group; 2005.

^11^ Alexopoulos G, Abrams R, Young R. Shamian C. Cornell scale for depression in dementia. Biol Psychol. 1998; 23: 271–284.

^12^ Cohen-Mansfield J, Werner P, Marx MS. Agitation Behaviour Mapping Instrument. An observational study of agitation in agitated nursing home residents. Int Psychogeriatr. 1989; 1(2): 153-165.

^13^ Lawton MP, Van Haitsma K. Klapper J. Lawton’s modified behaviour stream. observed affect in nursing home residents with Alzheimer’s disease. J Gerontol B Psychol Sci Soc Sci. 1996; 51B(1): 3-14.

^14^ van der Kam P, Mol F,Wimmers MFHG. (1971). *Beoordelingsschaal voor Oudere Patie¨nten*. Van Loghum Slaterus: Deventer. No English translation.

^15^ Lawton MP. the Philadelphia geriatric center morale scale: a revision. J Gerontol. 1975; 30(1): 85–89.

^16^ Goldberg DP (1978) *GHQ-28.* Manual of the general health questionnaire. Windsor: Nfer-Nelson.

^17^ van der Kooij CH. 1996. Validation en Belevingsgerichte Zorg. Deel 1: Op zoek naar gelijkwaardigheid. *Tijdschrift Voor Verzorgenden* 6: 26–31. No English translation.

^18^ EuroQol Group. EuroQol - EuroQolEQ-5D - a new facility for the measurement of health-related quality of

life. Health Policy. 1990; 16: 199-208.

^19^ Harwood RH, Ebrahim S. The tool and manual of the Short London Handicap Scale. University of Nottingham, 1995.

^20^ Mahoney F, Barthel D. Functional evaluation: The BARTHEL index. Md State Med J. 1965; 14: 61–65.

^21^ Cummings JL, Mega M, Gray K, Rosenberg-Thompson S, Carusi DA, Gornbein J. The Neuropsychiatric Inventory: comprehensive assessment of psychopathology in dementia. Neurol. 1994; 44: 2308–14.

^22^ Robinson B. Validation of a caregiver strain index. J Gerontol. 1983; 38: 344-348.

^23^ McCabe CJ, Thomas KJ, Brazier JE, Coleman P: **Measuring the mental health status of a population: a comparison of the GHQ-12 and the SF-36 (MHI-5).** Br J Psychiatry. 1996; **169:** 516–521.

^24^ Helmes E, Csapo KG, Short JA. Standardization and validation of the multidimensional observation scale for elderly subjects (MOSES). J Gerontol. 1987: 42(4): 395-405.

^25^ Lawton MP, Van Haitsma K, Klapper J. Apparent affect rating scale: observed affect in nursing home residents with Alzheimer’s disease. J Gerontol B Psychol Sci Soc Sci*.* 1996; 51B (1): 3-14.

^26^ Morris JN, Hawes C, Fries BE, Phillips CD, Mor, V, Katz S, et al. Designing the national resident assessment instrument for nursing homes [Gerontologist](https://www.researchgate.net/journal/0016-9013_The_Gerontologist). 1990; 30(3): 293-307.

^27^ Reisberg B, Borenstein J, Franssen E, Salob S, Steinberg G, Shulman, E, et al. (1987). BEHAVE-AD: A clinical rating scale for the assessment of pharmacologically remediable behavioral symptomatology in Alzheimer’s disease. In: H. Altman, H, editor. Alzheimer’s disease: problems, prospects, and perspectives. New York: Plenum Press; 1987. pp. 1-16**.**

^28.^ Teresi J, Lawton M P, Holmes T, & Ory, M. Measurement in elderly chronic care populations. New York: Springer Publishing

Company; 1997

^29^ Lawton M P, Brody E M (1969). Assessment of older people: self-maintaining and instrumental activities of daily living. Gerontologist. 1969; 9: 179-185.

^30^ Ancoli-Israel S, Cole R, Aless, C, Chambers M., Moorcroft W, Pollak C. The role of actigraphy in the study of sleep and circadian rhythms. Sleep. 2003: 26(3): 342–392.

^31^ Finkel SI, Lyons JS, Anderson RL. A brief agitation rating scale (BARS) for nursing home elderly. J Am Geriatr Soc. 1993; 41: 50–52.

^32^ Lyketsos CG, Galik E, Steele C, Steinberg M, Rosenblatt A, Warren A, Sheppard JM, et al. The general medical health rating: a bedside global rating of medical comorbidity in patients with dementia. J Am Geriatr Soc. 1999; 47: 487–491.

^33^ Ettema TP, Dröes, RM, deLange, J, Mellenbergh, GJ, Ribbe, MW. QUALIDEM: Development and evaluation of a dementia specific quality of life instrument– Validation. Int J Geriatr Psychiatry. 2007 22(5), 424-430

^34^ Landeweerd JA, Boumans NPG, Nissen JMF: Bedrijfsgezondheidszorg Studies nr. 11. De Maastrichtse arbeidssatisfactieschaal voor de gezondheidszorg (MASGZ). [*Industrial health care studies no. 11. The Maastricht Job Satisfaction Scale for Health Care (MJSS-HC)* In Dutch] Maastricht: University of Maastricht; 1996.
