# Supplementary material for: Effects of person-centered care at the organisational-level for people with dementia. A systematic review
Source: PLoS One. 2019 Feb 22;14(2):e0212686. doi: 10.1371/journal.pone.0212686 (PMC6386385; doi:10.1371/journal.pone.0212686)
Supplement: S1 Fig — (DOCX) [file pone.0212686.s003.docx]

**S1 Fig. MEDLINE search strategy**

1. exp Dementia/

2. Delirium, Dementia, Amnestic, Cognitive Disorders/

3. dement*.mp.

4. alzheimer*.mp.

5. (lewy* adj2 bod*).mp.

6. (chronic adj2 cerebrovascular).mp.

7. ('organic brain disease' or 'organic brain syndrome').mp.

8. (cerebr* adj2 deteriorat*).mp.

9. (cerebral* adj2 insufficient*).mp.

10. or/1-9

11. activity.ti,ab.

12. activities.ti,ab.

13. psychosocial.ti,ab.

14. non-pharmacological.ti,ab.

15. individually-tailor*.ti,ab.

16. personally-tailor*.ti,ab.

17. (individual or individuals or individually-cent*).ti,ab.

18. (meaning* OR meaningful*).ti,ab.

19. involvement.ti,ab.

20. (engagement or engaging).ti,ab.

21. occupational*.ti,ab.

22. personhood.ti,ab.

23. person-centred.ti,ab.

24. identity.ti,ab.

25. Personhood/ or Patient-Centred Care/

26. or/11-24

27. 10 and 26

28. randomized controlled trial.pt.

29. controlled clinical trial.pt.

30. randomly.ab.

31. groups.ab.

32. placebo.ti,ab.

33. randomi?ed.ti,ab.

34. ('double-blind*' or 'single-blind*').ti,ab.

35. (RCT or CCT).ti,ab.

36. or/28-35

37. 27 and 36
